# Patients satisfaction with laboratory services at antiretroviral therapy clinics in public hospitals, Addis Ababa, Ethiopia

**DOI:** 10.1186/1756-0500-5-184

**Published:** 2012-07-04

**Authors:** Tedla Mindaye, Bineyam Taye

**Affiliations:** 1 College of Health Science, School of Clinical laboratory Science, Addis Ababa University, Addis Ababa, Ethiopia; 2 College of Health Science, School of public health, Addis Ababa University, Addis Ababa, Ethiopia

**Keywords:** Patient satisfaction, ART, Laboratory services, Ethiopia

## Abstract

**Background:**

Despite the fact that Ethiopia has scale up antiretroviral treatment (ART) program, little is known about the patient satisfaction with ART monitoring laboratory services in health facilities. We therefore aimed to assess patient satisfaction with laboratory services at ART clinics in public hospitals.

**Methods:**

Hospital based, descriptive cross sectional study was conducted from October to November 2010 among clients attending in nine public hospitals ART clinics in Addis Ababa Ethiopia. Patients’ satisfaction towards laboratory services was assessed using exit interview structured questionnaire. Data were coded and entered using EPI info 2002 (Centers for Disease Control and Prevention Atlanta, GA) and analyzed using SPSS version 15 software (SPSS INC, Chicago, IL, USA).

**Results:**

A total of 406 clients were involved in the study. Of these 255(62.8%) were females. The overall satisfaction rate for ART monitoring laboratory services was (85.5%). Patients were satisfied with measures taken by health care providers to keep confidentiality and ability of the person drawing blood to answer question (98.3% and 96.3% respectively). Moreover, the finding of this study revealed, statistical significant associations between the overall patients’ satisfaction with waiting time to get blood drawing service, availability of ordered laboratory tests and waiting time to get laboratory result with (p < 0.05). Patients receiving blood drawing service less than 30 minute were 7.59 times (95% CI AOR: 3.92–14.70) to be more satisfied with ART monitoring laboratory services compared to those who underwent for more than 30 minutes.

**Conclusions:**

Overall, the satisfaction survey showed, most respondents were satisfied with ART monitoring laboratory services. However, factors such as improving accessibility and availability of latrines should be taken into consideration in order to improve the overall satisfaction.

## Background

Patient satisfaction is the patient’s perception of care received compared with the care expected [1]. Evaluating to what extent patients are satisfied with health services is clinically relevant, as satisfied patients are more likely to comply with treatment [2], take an active role in their own care [3], continue using medical care services and stay within a health provider (where there are some choices) and maintain with a specific system [2]. On the other hand clients who are not satisfied with a service may have worse outcomes than others because they miss more appointments, live against advice or fail to follow through on treatment plans.

In clinical laboratory monitoring patients satisfaction is an important and useful tools required for quality improvement as well as to maintain their accreditation [4,5].

Antiretroviral treatment (ART) monitoring laboratory services have crucial roles in delivery of quality of ART by diagnosing and staging HIV infection [6]. However, comprehensive quality laboratory services are a challenging process; need multiple sources of supports from clients, providers, managers, and other stakeholders. Especially, needs and preferences of clients in clinical laboratory must be addressed in the design and implementation of laboratory quality system. Disregard for patients’ feedback may cause persistent disruption of testing because a patient has to return several times for the results and treatment. Thus monitoring patient satisfaction is an important and useful quality improvement indicator and is required by clinical laboratories [4].

The literature indicates that there are only a few reports of patients’ satisfaction from developing countries, as compared to the high volume of publications on patients’ satisfaction from developed countries [7,8]. In Ethiopia, most patients’ satisfaction survey are carried out in general health service either outpatient or inpatient departments [9-12]. However, to our knowledge there is no study conducted particularly in ART monitoring laboratory services.

Therefore, this study was designed to assess patient satisfaction to ward ART monitoring laboratory services in public hospitals of Addis Ababa, Ethiopia.

## Methods

### Study setting and context

Hospital based, descriptive cross sectional study was conducted between October and November 2010. The study was conducted in Addis Ababa which is the capital city of Ethiopia. Addis Ababa has a population size of 2,738,248 million with annual growth rate of 2.1 [13]. The city is divided into ten sub-cities and 99 kebeles (Lowest level administrative unit in the city). This study focused on nine public hospitals which have been providing antiretroviral treatment services for people living with HIV/AIDS (PLWHA). The hospitals were Tikur Anbessa specialized teaching hospital, St. Peters TB specialized hospital, ALERT, St. Paul, Zewditu Memorial, Yekatit 12, Minilik II, Ras Desta Damtew, and Ghandi memorial hospitals.

The client load of ART clinics in each hospital according to the Federal HIV/AIDS Prevention and Control Office (FHAPCO) monthly HIV care and ART update as of February, 2010 was 2619 for Tikur Anbessa specialized teaching hospital, 1769 St. Peters TB specialized 4945 ALERT, 3170 St. Paul, 5685 Zewditu Memorial, 2146 Yekatit 12, 1758 Minilik II, 1008 Ras Desta Damtew, and 238 Ghandi memorial hospitals [14]. Each hospital has a separate laboratory units specifically designed to serve only HIV infected patients enrolled in ART clinic and perform laboratory tests used to monitoring ART follow up. The service render in these laboratories are ART monitoring tests including clinical chemistry (liver and renal function tests), hematology (Complete Blood Count, CD4 count) and other laboratory tests used for the management of HIV infected taking ART. Patients visit laboratory every 3 months and had proper counseling about drug adherence and laboratory test used for monitoring their treatment.

### Study subjects

Adult PLWHA (>18 years of age) who visited ART clinics for at least three months and referred to ART clinics laboratory for ART monitoring laboratory tests including clinical chemistry (liver and renal function), Hematology (Complete Blood Count, CD4 count) and were not confused or too ill to participate.

### Sample size and sampling producer

The sample size was estimated based on the assumption that 50% of the patients attending in ART clinic laboratories are satisfied, a 5% margin of error, and a 95% confidence level (P = 50% was taken due to absence of reliable previous study that show patents satisfaction ART monitoring laboratory service in Ethiopia). The initial sample size was 384 however, considering 10% non response rate the final sample size was 422.

The sample size in each public hospital was subsequently determined according to the proportion of the client load of ART clinics in each hospital after reviewing the monthly HIV care and ART update as of February, 2010 by FHAPCO/MOH [14]. Adult patients were randomly selected from those attending ART clinics in each hospital using ART clinic registration books as the sampling frame for selection of patients.

### Measurement and data collection

The data was collected via face-to-face interviews, in which the translated Patients’ Satisfaction Questionnaire in Amharic was used to guide the researcher. All interviews were carried out by one of the investigators to standardize interviews and reduce interview biases.

The questionnaire contains of satisfaction indicators which are related to socio-demographic characteristics of the patients and different dimensions of ART monitoring laboratory services such as waiting time, availability of requested laboratory tests, needle stick attempt, information provision about bruise, availability of space in blood drawing room, privacy, respect and courtesy, confidentiality.

Standardized 5-point Likert scales ranging from strongly disagree to strongly agree (1 to 5 points) were used for all items. Patients’ satisfaction were classified; into two categories satisfied and dissatisfied by using the demarcation threshold from formula: {(total highest score-total lowest score)/2} + Total lowest score [15].

### Statistical analysis

Data were coded and entered using EPI info 2002 (Centre for Disease Control and Prevention Atlanta, GA) and analyzed using SPSS version 15 software (SPSS INC, Chicago, IL, USA). Descriptive statistics were computed to ascertain the percentage of patient Satisfaction in each satisfaction indicators. Binary and multiple logistic regressions were subsequently conducted to predict the factors which influence the level of satisfaction with ART monitoring laboratory services. P-Value less than 0.05 were taken as statistically significant.

### Ethical consideration

Before any attempt to collect data, approval to conduct the study was obtained from Institutional Review Board (IRB) of School of Medicine, Addis Abba University. Each participant (patient) was notified about the purpose of the study, the right to refuse to participate in the study, and anonymity and confidentiality of the information gathered. They were assured that they would not be penalized for not participating if they wished not to participate, and that their responses to the questions would have no effect on their care.

## Results

A total of 422 respondents were selected as the sample of the study. However, 16 respondents (3.8%) refused to participate, and hence, 406 clients of the antiretroviral clinics in nine public hospitals were interviewed in this study. The response rate derived in this study was 96.2%.

Based on the result of this study that majority of respondents were females 255(62.8%), age group 28–37 204(50.2%); with family monthly income 500–1000 Ethiopian Birr (1ET.Birr = 17.2USD). Also, this table showed that most of the respondents had attended at least secondary school education 208(51.2%) and employee of private sectors 140(34.5%) (Table 1).

**Table 1:** Socio demographic characteristics of patients of Antiretroviral Treatment service in the public hospitals, in Addis Ababa, December, 2010 (n = 406)

**Variable**	**Number**	**Percentage (%)**
**Sex**		
Male	151	37.2%
Female	255	62.8%
Total	406	100.0%
**Age**		
18–27	46	11.3%
28–37	204	50.2%
38–47	96	23.6%
48–57	44	10.8%
58 & above	16	3.9%
Total	406	100.0%
**Marital status**		
Single	110	27.1%
Married	151	37.2%
Divorced	63	15.5%
Widowed	82	20.2%
Total	406	100.0%
**Religion**		
Orthodox	312	76.8%
Muslim	38	9.4%
protestant	54	13.3%
Others	1	0.2%
no answer	1	0.2%
Total	406	100.0%
**Educational status**		
Illiterate	36	8.9%
Read and write	42	10.3%
Primary school(1–8)	73	18.0%
Secondary school completed	208	51.2%
College	47	11.6%
Total	406	100.0%
**Occupation**		
Student	10	2.5%
Government employee	68	16.7%
Private employee	140	34.5%
Daily laborer	46	11.3%
Merchant	24	5.9%
Housewife	45	11.1%
Unemployed	73	18.0%
Total	406	100.0%
**Income class in Eth Birr**^**1**^		
Less than 150	98	24.1%
150–499	71	17.5%
500–1000	141	34.7%
over 1000	49	12.1%
Non response	47	11.6%
Total	406	100.0%

^1^ Eth Birr equivalent to 17.2 USD.

### Level of satisfaction of clients with laboratory service

The result regarding respondents’ satisfaction with different dimensions of ART monitoring laboratory services are presented in Table 2. Generally, most of the respondents were satisfied with the providers’ courtesy/respect 382(94.1%), cleanliness of blood drawing area 386(95.6%), measures taken by health care providers to keep confidentiality 399(98.3%), completeness of information when and how client receive laboratory results 376(92.6%), ability of the person drawing blood to answer questions 391(96.3%) and comfort of chairs in blood drawing room 361(88.1%). On the other hand most of the clients showed low satisfaction level with latrine cleanness and comfort 257(63.5.0%), accessibility and availability of latrines 262(64.5%).

**Table 2:** Level of satisfaction of clients of ART clinic in the public hospitals of Addis Ababa, December, 2010 (n = 406)

	**Level of Satisfaction**	
**Variables**	**Number**	**%**
Privacy during blood drawing	362	89.2
Cleanliness of blood drawing area	386	95.6
Respect and courtesy	382	94.1
Ability of Person drawing blood to put client at ease	382	94.1
Comfort of chairs	361	88.9
Completeness of information on how and when to receive lab result	376	92.6
Ability of the person drawing blood to answer question	391	96.3
Latrine accessibility and availability	262	64.5
Latrine cleanness and comfort	257	63.5
Cleanness and comfort of waiting area	363	89.4
Confidentiality measure	399	98.3
Overall service satisfaction *	347	85.5

* Based on this formula (Total highest score-total lowest score)/2} + Total lowest score), satisfaction level threshold was set at the score > 33.

In overall, a vast majority of the respondents 347 (85.5%) were satisfied with ART monitoring laboratory service received at nine public hospital in Addis Ababa (Table 2).

### The relationship between the patients’ level of satisfaction and independent variables

The chi-square of independence was conducted to assess whether the level of patients’ satisfaction had a relationship with explanatory variables.

The results from the cross-tabulations analysis showed that there were no significant relationship between gender, Educational background, marital status, occupational status, and family monthly income with level of patients’ satisfaction toward ART monitoring laboratory services (P value > 0.05). However, there were significant relationship between age group with level of patients’ satisfaction (*x*^2^ = 8.10, df = 3, p = 0.04, n = 344). Furthermore, there was significant relationship between availability of place for blood drawing room to put things, Information provision about bruise, availability of lab tests, Waiting time to get blood drawing service and laboratory results with level patients’ satisfaction toward ART monitoring laboratory service (P value <0.05) (Table 3).

**Table 3:** Relationship between level of Patients’ satisfaction with independent variable (n = 406)

**Variables**	**Satisfied (n)**	**Not Satisfied** (n)	***X***^**2**^	**df**	P value
Gender					
Male	127	24	0.072	1	
Female	217	38			0.788
Age Group					
18–27	42	4			
28–37	163	40			
38–47	83	14	8.10	3	
48–57	56	4			0.04
Educational status					
Illiterate	34	2			
Read and write	36	6	3.16	4	
Primary school(1–8)	62	11			0.531
Secondary school completed	173	35			
Collage	39	8			
Marital status					
Single	91	19			
Married	130	21	1.08	3	
Divorced	55	8			0.780
Widowed	68	14			
Occupation					
Unemployed	178	33			
Employee	166	29	0.046	1	
Income class					0.891
Less than 150	85	13			
150–499	64	7	5.23	3	
500–1000	118	23			0.155
over 1000	37	12			
Availability of place in blood drawing					
Yes	71	3			
No	272	59	8.84	1	
Information provision about bruise					0.00
Yes	180	20			
No	164	42	7.68	1	
Availability of lab tests					0.00
Yes some only	109	27			
Yes all	233	35	12.9	1	
Waiting time to get blood drawing service					0.00
<30 minutes	253	15			
½–1 hour	36	10			
1–2hours	31	22	65.28	3	
>2 hrs	24	15			0.00
No of Needle stick attempt					
One veni puncture	268	46			
Two veni puncture	65	11	3.28	2	
Three veni puncture	11	5			0.19
Waiting time to get lab result					
<1 hours	74	7			
1–2 hour	27	10	18.2	2	
>2 hours	243	45			0.00

### Factors affecting the level of clients’ satisfaction

In univarate analysis, overall satisfaction of clients toward ART monitoring laboratory services showed statistically significant association with waiting time to get blood drawing service, availability of place in blood drawing room to put client personal things, provision of information regarding bruise due to blood drawing, availability of ordered laboratory tests (p < 0.05).

When adjusted odds ratios were calculated statistical significant associations were found between the overall satisfaction of the clients with waiting time to get blood drawing service, availability of ordered laboratory tests and waiting time to get laboratory result with (p < 0.05).

Results of multiple logistic regression analysis showed that clients who waited less than 30 minutes to get blood drawing services were 7.5 times more likely to be satisfied than those who waited more than 30 minutes (AOR = 7.59: CI: 3.92–14.70). Moreover those clients who waited less than two hours to get laboratory results were five times more likely to be satisfied than those who waited more than two hours (AOR = 5.52; CI: 1.58–19.23) and clients who got all requested laboratory tests were 2.3 times likely to be satisfied than those who did not got the information (AOR = 2.36;CI: 1.26–4.44) (Table 4).

**Table 4:** Predictor variables of the level of patients’ satisfaction toward ART monitoring laboratory services

**Variables**	**Dependent Variable**		**Crude odds ratio (95% CI)**	**P value**	**Adjusted OR(95% CI)**	**P value**
		Sat(n)	Sat.(n)				
**Availability of place in blood drawing room to put things**						
No	271	59	1		1	
Yes	71	3	5.13 (1.56–16.8)*	0.007	2.02(0.56–7.34)	0.282
**Information provision about bruise**						
No	164	42	1		1	
Yes	180	20	2.30(1.30–4.0)*	0.004	1.81(0.93–3.51)	0.077
**Availability of lab tests**						
Yes some only	109	27	1		1	
Yes all	233	35	2.72(1.56–4.71)*	0.000	2.36 (1.26–4.44)*	0.007
**Waiting time to get blood drawing service**						
>30 minutes	253	15	1		1	
<30 minutes	91	47	8.71(4.64–16.3)*	0.000	7.59 (3.92–14.70)*	0.000
**Number of Needle stick attempts**						
More than one attempts	76	16	1		1	
Only One attempt	268	46	0.81 (0.43–1.52)	0.521	1.28(0.60–2.71)	0.511
**Waiting time to get lab result**						
>2 hours	243	59	1		1	
< 2 hours	101	3	8.17(2.50–26.60)*	0.000	5.52(1.58–19.23)*	0.007

*Significant Associations as P < 0.05,

## Discussion

This study has revealed that overall client satisfaction level with the ART monitoring laboratory services were 85.5% that showed a vast majority of the respondents were satisfied with almost all aspects of ART monitoring laboratory services they received. The results reported here could be explained in several ways. One explanation is that the structure of the questionnaire was limited in indicating underlying factors. On the other hand, the high satisfaction could be due to introduction of social desirability biases by clients. Clients might not be ready to tell their dissatisfaction status freely since the interviews were carried out within the hospitals. Again, it should be remembered that, unless special precautions are taken, clients may be reluctant to reveal their opinions for fear of alienating their attendants as ART monitoring laboratory services are given free of charge [3]. A similar study conducted by Akhtari-Zavare M et al. in government teaching hospitals in Tehran, they found that 82% of these patients were satisfied [16].

The result of the present study has also showed higher patient satisfaction rate than the reports by other researchers in Ethiopia as shown 57.1% and 43.6%, in Jimma and Tigray respectively [17,18]. The underlying justifications for higher clients’ satisfaction with ART services than outpatient services in other areas include multiple factors. ART services in Ethiopia are focus of attentions for government and many donors. Different interventions of monitoring, reporting including policies and implementation guidelines are functional for ART services. Many Donors are investing on the program large amount of resources and give technical supports. Clients are also benefiting from improved quality of life, decreased morbidities and mortality due to ART.

Mfinanga SG et al. [19] reported that patients with higher education were more likely to be dissatisfied. On the contrary, results of this study showed that there was no relationship between the educational status of the patients and their overall satisfaction with ART monitoring laboratory service. This could be due to the fact that most of patients were completing only secondary school; hence there was no difference in their expectations.

The result of this study showed a significant relationship between waiting time to get blood drawing service and laboratory results with level patients’ satisfaction toward ART monitoring laboratory service which is consistent with the study conducted in Amhara and Tigray region showing that long waiting hours were associated with dissatisfaction [9,18].

Based on the findings, the patients receiving blood drawing service less than 30 minute were found to be more satisfied with the ART monitoring laboratory services compared to those who underwent for more than 30 minutes. The results of this study showed that there was a significant relationship between the level of satisfaction and the availability of the requested laboratory tests during their visit in the laboratory. One possible explanation for the significant finding is that patients who fail to get the requested laboratory tests at the time could have been forced to get the laboratory service at private facility with high costs. Such event usually happened when laboratory equipments such as CD4 machine could have been broken down; as a consequent, they had to wait for the hospitals to get the machine repaired.

Logistic regression showed that waiting time to get blood drawing service and waiting time to get laboratory results are the predictor for level of patients’ satisfaction. This result is similar with the study conducted by Soleimanpour et al., on patients hospitalized at emergency department the results showed that; overall satisfaction rate was dependent on the mean waiting time [20].

### Limitation

There are several limitations to the design of this study. One limitation is related to the selection bias. This was due to the fact that the participation in the study was based on voluntary basis, usually those voluntary are satisfied and don’t have complain from ART monitoring laboratory services. Consequently increase the result of satisfaction from ART monitoring services. On the other hand, Clients of ART might give biased information since interview was conducted in the hospitals. Furthermore a sample of 406 patients is not big enough to detect any significant association between socio-demographic characteristics and level of patients’ satisfaction.

## Conclusions

Overall, the satisfaction survey showed that most respondents were satisfied with ART monitoring laboratory services. However, the respondents suggested that several factors such as improving accessibility and availability of latrines should be taken into consideration in order to improve the overall satisfaction.

Also, higher level of measure taken to keep confidentiality, ability of the person drawing blood to answer question and cleanliness of blood drawing area may increase patients’ level of satisfaction towards ART monitoring laboratory services.

Finally, this study also showed that the patients, receiving blood drawing service less than 30 minute, were found to be more satisfied with the ART monitoring laboratory services compared to those who underwent for more than 30 minutes. Further improvement can also be achieved, if ART monitoring laboratories institutionalizing quality management system and using its feed back in a systematic way to enhance patient satisfaction with ART monitoring laboratory services.

## Authors’ contributions

TM conceived and designed the study and collected data, performed analysis, Interpretation of data, and draft the manuscript. BT assisted with the design, performed analysis, interpretation of data and the critical review of the manuscript. All authors read and approved the final manuscript. All authors participated in critical appraisal and revision of the manuscript.

## Competing interests

All authors declare that they have no conflict of interest associated with the publication of this manuscript.
